# Higher Soluble ACE2 Levels and Increased Risk of Infection-Related Hospitalization in Patients on Maintenance Hemodialysis

**DOI:** 10.3389/fmed.2022.791284

**Published:** 2022-01-26

**Authors:** Mayuko Kawabe, Akio Nakashima, Izumi Yamamoto, Ichiro Ohkido, Takashi Yokoo, Mitsuyoshi Urashima

**Affiliations:** ^1^Division of Molecular Epidemiology, The Jikei University School of Medicine, Tokyo, Japan; ^2^Division of Nephrology and Hypertension, Department of Internal Medicine, The Jikei University School of Medicine, Tokyo, Japan

**Keywords:** angiotensin-converting enzyme 2, soluble ACE2, infection, all-cause death, maintenance hemodialysis

## Abstract

**Background:**

Angiotensin-converting enzyme 2 (ACE2) works as an endogenous counter-regulator of the renin-angiotensin system, which has pivotal roles in preventing both cardiovascular disease (CVD) and inflammation. In general populations, higher plasma soluble ACE2 levels were reported to be associated with increased risks of all-cause death and major CVD. Because infections are fatal in patients on maintenance hemodialysis, we aimed to explore whether soluble ACE2 levels are associated with an increased risk of infection-related hospitalization in these patients.

**Methods:**

Using data from a prospective, multicenter, cohort study conducted in Tokyo, Japan, we performed a *post-hoc* analyses of 724 clinically stable patients on maintenance hemodialysis. We measured baseline serum soluble ACE2 levels and assessed potential determinants of its with infection-related hospitalization as a primary outcome as well as all-cause death and CVD as secondary outcomes using a Cox proportional hazards model.

**Results:**

The soluble ACE2 level (median, 0.16 ng/ml; interquartile range, 0.07–0.57 ng/ml) showed a weak negative association with age. During a median follow-up of 39 months, 106 patients (14.6%) were hospitalized with infectious diseases. Compared with the lower half of soluble ACE2 levels, the higher half was associated with an increased risk of infection-related hospitalization (hazard ratio, 1.57; 95% confidence interval, 1.02–2.41) with adjustment by other risk factors. On the other hand, there were no significant associations between soluble ACE2 and risks of all-cause death and CVD.

**Conclusion:**

Higher soluble ACE2 levels may associate with an increased risk of infection-related hospitalization in patients on maintenance hemodialysis.

## Introduction

Angiotensin-converting enzyme 2 (ACE2) works as an endogenous counter-regulator of the renin-angiotensin system through angiotensin II cleavage, which has pivotal roles in preventing both cardiovascular disease (CVD) and inflammation (1–3). ACE2 is a transmembrane protein (mACE2), which is shed into the blood circulation as its soluble form (sACE2) through proteolytic cleavage by ADAM Metallopeptidase Domain 17 (ADAM17) (4–6). A recent large observational study reported that increased plasma sACE2 levels were associated with increased risks of major CVD events and all-cause death in general populations (7). However, associations of serum sACE2 levels with infectious diseases accompanied by severe inflammation have not yet been examined.

In patients with chronic kidney disease (CKD), evidence suggests that uremia is associated with a worse immune response (8–10). In addition, patients on long-term hemodialysis (HD) are well-known to be vulnerable to infectious diseases (11, 12) and at high risk of developing coronavirus disease 2019 (COVID-19), with a high mortality rate (13, 14). Therefore, by conducting *post-hoc* analyses of a prospective cohort study (15–17), the aim was to explore whether serum levels of sACE2 are associated with infectious diseases requiring hospitalization as a primary outcome, as well as all-cause death and CVD as secondary outcomes, in patients on maintenance HD.

## Materials and Methods

### Study Design and Cohort

Previously, a prospective, multicenter, cohort study was conducted by enrolling HD patients who were recruited at the dialysis outpatient units of 15 medical institutions in Tokyo, Japan, between May 1, 2011 and March 31, 2012. The patients were followed-up until June 1, 2015. All patients were 20 years or older, had spent at least 3 months on dialysis therapy, and regularly received thrice-weekly HD (3–5 h/session). Patients were excluded if they had acute gastrointestinal bleeding, acute coronary syndrome, liver dysfunction, active infection or were prescribed antibiotics at baseline enrollment.

Using data from this prospective, multicenter, cohort study, *post-hoc* analyses of 724 patients with sufficient volumes of serum samples were conducted. Some cases were involved in previous studies using the same database (15–17). The ethics committee of the Jikei University School of Medicine [ethics approval code: 22-182(6359)] approved the study protocol. This prospective, cohort study was also approved by each participating institution's review board. Written, informed consent was obtained from all patients prior to inclusion in the study. All study procedures were in accordance with the Declaration of Helsinki and its revisions.

### Data Collection

Age, sex, dialysis vintage, body mass index (BMI), primary kidney disease leading to HD, and past medical history were extracted from medical records. Medication information was obtained from prescription records. Comorbidities and medications were determined by chart review and standardized interviews at baseline. Blood samples were collected at study entry, before the HD session after the longest inter-dialysis period. Routine biochemical measurements included serum albumin, blood urea nitrogen, creatinine, sodium, potassium, calcium, phosphate, and C-reactive protein levels as well as complete blood count. The serum samples were stored at −80°C prior to use.

### Measurement of Serum ACE2 Levels

Serum ACE2 levels were measured using a human ACE2 enzyme-linked immunosorbent assay (ELISA) kit (#ELH-ACE2-1, RayBiotech, Peachtree Corners, GA, USA) in accordance with the manufacturer's instructions by a study investigator (M.K.). The molecular weight measured using the human ACE2 ELISA kit is 83.6 kDa. All serum samples were measured by the same lot number. First, all samples were measured without dilution; 656 samples did not need dilution, and 68 samples were diluted 1:10 with the supplied solution before being added to the wells. Five of 68 samples needed five-fold or 50-fold dilutions according to the lower or upper limit of the standard range. Subgroups were stratified by the median level into either low or high sACE2 level groups. The measurer was blinded to the clinical and biochemical data of the study.

### Measurement of IL-6 and sIL-6R Levels

Serum interleukin-6 (IL-6) levels were measured using a Quantikine® HS Human IL-6 Immunoassay ELISA kit (#HS600C, R&D Systems, Minneapolis, MN, USA), and soluble interleukin-6 receptor (sIL-6R) levels were measured using a Human IL-6 sR ELISA kit (#ELH-IL6sR, RayBiotech) in accordance with the manufacturer's instructions by a study investigator (M.K.). Serum IL-6 levels were measured in 639 samples, and sIL-6R levels were measured in 699 samples because the residual volumes of the serum samples were not sufficient. IL-6 levels were measured with four-fold dilutions, and 17 of 639 samples needed 10-fold, 20-fold, or 100-fold dilutions according to the upper limit of the standard range. The sIL-6R levels were measured with 500-fold dilutions for all 699 samples. Both the IL-6 and sIL-6R subgroups were stratified by the median level. The measurer was blinded to the clinical and biochemical data of the study.

### Outcomes

Clinical outcomes of infection-related hospitalization, all-cause death, and CVD were prospectively recorded and coded, blinded from clinical and biochemical data. This information was collected by a different study investigator (A.N.). The primary outcome was infection-related hospitalization. The secondary outcomes were all-cause death and CVD. Infection-related hospitalization was defined as a composite of death due to infectious diseases and the first event requiring hospitalization. For each patient, only the first event, but not repeated events, was counted in the analysis. Censoring occurred at the time of kidney transplantation, at loss of follow-up, or at the end of the study.

### Statistical Analysis

Spearman's rank correlation coefficient (rho) was used to quantity the strengths of associations between two continuous variables: rho ≥ 0.9, very strong; 0.9 > rho ≥ 0.7, strong; 0.7 > rho ≥ 0.4, moderate; 0.4 > rho ≥ 0.1, weak; and rho <0.1, negligible (18). To determine if sACE2 levels were associated with clinical and biochemical parameters, linear regression was performed for each variable. Continuous variables with normal distributions and with non-normal distributions were compared by the unpaired *t*-test and the Mann-Whitney test, respectively. Dichotomous variables were compared between the two groups by the chi-squared test. Data are presented as means and standard deviation (SD) for continuous variables with normal distributions or medians and interquartile range (IQR) for the continuous variables with non-normal distributions. The follow-up period was aggregated on a monthly basis, so a follow-up period of <1 month was replaced with 0.5 months for the analysis. The effects of sACE2 levels on infection-related hospitalization, as well as all-cause death and CVD, were analyzed using Nelson-Aalen cumulative hazard curves for outcomes by a study investigator (M.U.) and confirmed by another investigator (M.K.). A Cox proportional hazards model was used to determine hazard ratios (HRs) and 95% confidence intervals (CIs). To confirm the robustness of the results, three distinct Cox proportional hazards models were used as a sensitivity analysis. The HRs were calculated by univariate analysis and multivariate analysis adjusted for sACE2, age, sex, dialysis vintage, BMI, diabetes mellitus in model 1 and model 1 plus hemoglobin, albumin, creatinine, Kt/V, IL-6, and sIL-6R in model 2. For model 3, the following variables were included: model 2 plus past history of CVD, past history of malignancy, use of angiotensin-converting enzyme inhibitor (ACE-I) or angiotensin receptor blocker (ARB), and use of β blocker. Multiple imputation was not performed because there were no missing data in primary and secondary outcomes as well as in sACE2 values. Values with two-sided *p* <0.05 were considered significant. All data were analyzed using Stata 15.0 (StataCorp LP, College Station, TX, USA).

## Results

### Study Population

**Figure 1:**
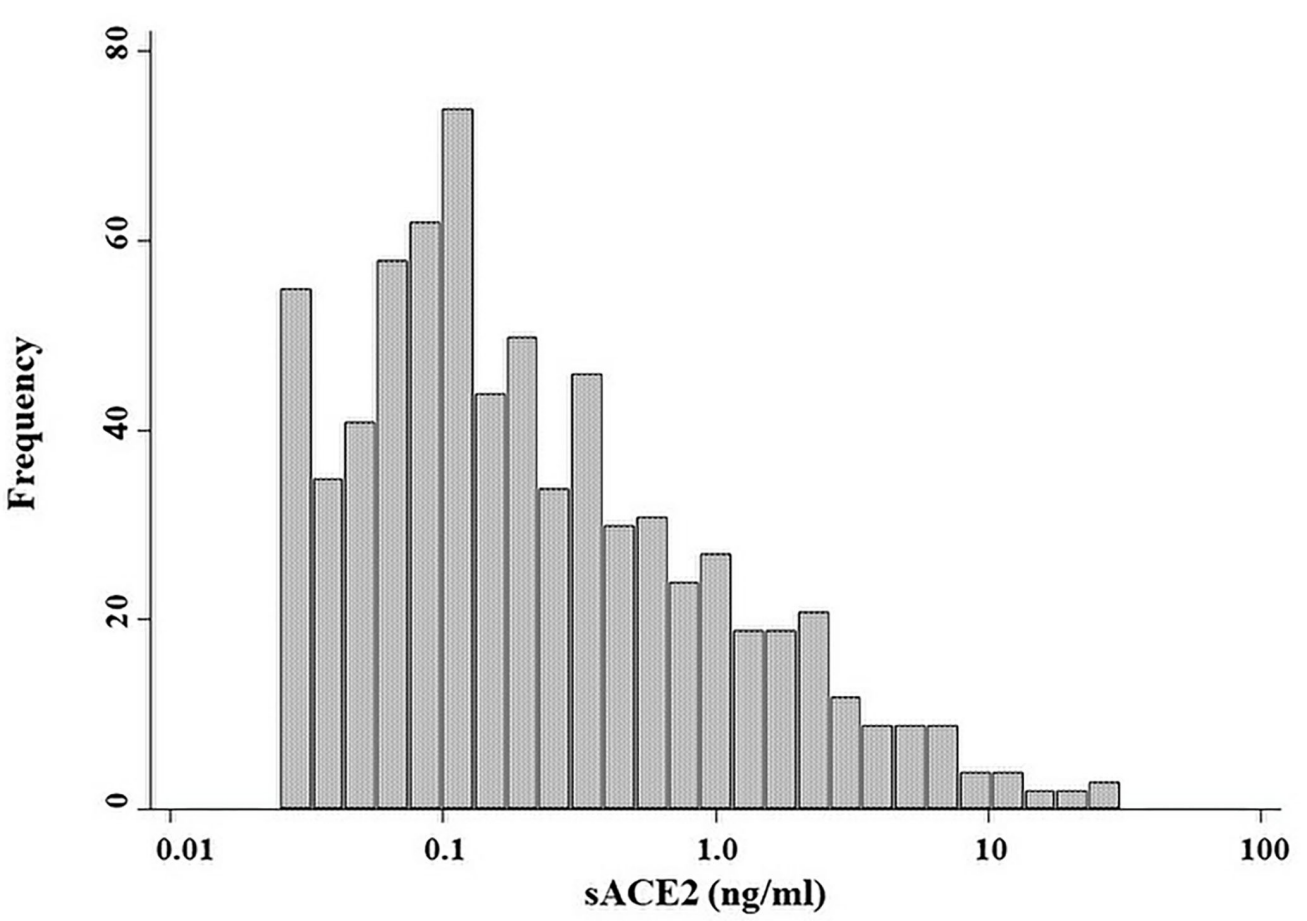
Distribution of serum ACE2 levels. Histogram shows logarithm-transformed levels of serum ACE2 for all patients.

A total of 724 patients were included in this study. The median age and dialysis vintage were 64 years (IQR, 55–72) and 6.9 years (IQR, 3.3–12.2), respectively. Male patients accounted for 70% of all patients. As the primary kidney disease, diabetes mellitus was the most frequent (35.8%). ACE-Is or ARBs were taken by 345 (49.4%) patients. Serum sACE2 levels ranged from undetectable (<0.025 ng/ml) in 37 patients (5.1%) to a maximum of 30.5 ng/ml; the median was 0.16 ng/ml (IQR, 0.07–0.57 ng/ml) (Figure 1). The baseline characteristics of the two subgroups divided by the median value of sACE2 are shown in Table 1. Between the two subgroups, there were no differences in patients' characteristics except that the age of patients in the higher half of sACE2 was younger than that of the lower half. The median follow-up periods were 39 months.

**Table 1:** Clinical characteristics of patients undergoing hemodialysis by sACE2 levels.

**Variable**	**Lower half of sACE2 (<0.16 ng/ml)**	**Higher half of sACE2 (0.16–30.54 ng/ml)**	**Total *N* = 724**
	***N* = 363**	***N* = 361**	
Age, y	65 (58–72)	63 (52–71)	64 (55–72)
Sex (male), *n* (%)	252 (69.4)	255 (70.6)	507 (70.0)
Dialysis vintage, y	7.5 (3.3–12.8)	6.7 (3.3–11.5)	6.9 (3.3–12.2)
Body mass index, kg/m^2^	21.4 (19.3–23.9)	21.5 (19.4–24.8)	21.5 (19.3–24.3)
Systolic blood pressure, mmHg*	150 ± 23	152 ± 22	151 ± 22
Diastolic blood pressure, mmHg	78 (70–87)	80 (70–90)	80 (70–90)
Primary kidney disease			
Diabetes mellitus, *n* (%)	130 (35.8)	129 (35.7)	259 (35.8)
Nephrosclerosis, *n* (%)	46 (12.7)	34 (9.4)	80 (11.1)
IgA nephropathy, *n* (%)	23 (6.3)	24 (6.7)	47 (6.5)
Chronic glomerulonephritis, *n* (%)	69 (19.0)	58 (16.1)	127 (17.5)
Polycystic kidney disease, *n* (%)	20 (5.5)	24 (6.7)	44 (6.1)
Others, *n* (%)	40 (11.0)	40 (11.1)	80 (11.1)
Unknown, *n* (%)	35 (9.6)	52 (14.4)	87 (12.0)
Diabetes mellitus, *n* (%)	134 (36.9)	138 (38.2)	272 (37.6)
Statin, *n* (%)	102 (28.1)	103 (28.5)	205 (28.3)
ACE-I or ARB, *n* (%)	174 (49.3)	171 (49.4)	345 (49.4)
β blocker, *n* (%)	92 (25.3)	94 (26.0)	186 (25.7)
Ca blocker, *n* (%)	135 (37.2)	150 (41.6)	285 (39.4)
α blocker, *n* (%)	31 (8.5)	22 (6.1)	53 (7.3)
Past history			
Cardiovascular disease, *n* (%)	69 (19.0)	66 (18.3)	135 (18.7)
Malignancy, *n* (%)	68 (18.7)	51 (14.1)	119 (16.4)
Fracture, *n* (%)	34 (9.4)	21 (5.8)	55 (7.6)
Hemoglobin, g/dl	10.4 (9.8–10.9)	10.5 (9.8–11.0)	10.4 (9.8–11.0)
Albumin, g/dl*	3.7 ± 0.3	3.7 ± 0.4	3.7 ± 0.4
Blood urea nitrogen, mg/dl*	64 ± 14	66 ± 14	65 ± 14
Creatinine, mg/dl	11.3 (9.6–13.5)	11.9 (9.7–13.8)	11.6 (9.7–13.7)
Kt/V	1.4 (1.2–1.5)	1.4 (1.2–1.5)	1.4 (1.2–1.5)
Sodium, mEq/l	139 (137–141)	139 (137–141)	139 (137–141)
Potassium, mEq/l	4.9 (4.5–5.3)	5.0 (4.5–5.4)	5.0 (4.5–5.4)
Calcium, mg/dl	8.9 (8.5–9.3)	8.8 (8.4–9.3)	8.9 (8.5–9.3)
Phosphate, mg/dl	5.4 (4.6–6.3)	5.5 (4.7–6.3)	5.4 (4.7–6.3)
C-reactive protein, mg/dl	0.13 (0.06–0.35)	0.14 (0.05–0.53)	0.13 (0.05–0.41)
Interleukin-6, pg/ml	5.3 (3.4–9.5)	6.0 (3.2–9.5)	5.6 (3.3–9.5)
Soluble interleukin-6 receptor, ng/ml	28.9 (20.0–40.3)	29.6 (20.1–39.4)	29.2 (20.0–40.0)

*sACE2, soluble angiotensin-converting enzyme 2; ACE-I, angiotensin-converting enzyme inhibitor; ARB, angiotensin II receptor blocker.*

Values are n (%) or presented as means ± standard deviation or medians (interquartile range, 25th−75th percentiles). *Unpaired t-test.

### Associations Between sACE2 Levels and Other Covariates

On univariate linear regression analysis, age, serum blood urea nitrogen, creatinine, and past history of malignancy were associated with logarithm-transformed sACE2 levels. A weak negative association was observed between age and logarithm-transformed sACE2 levels (rho = −0.16), and the others were negligible. There were no significant associations between serum sACE2 levels and sex, dialysis vintage, BMI, diabetes mellitus, past history of CVD, IL-6, sIL-6R, and use of ACE-Is or ARBs.

### Associations Between sACE2 Levels and Infection-Related Hospitalization

**Figure 2:**
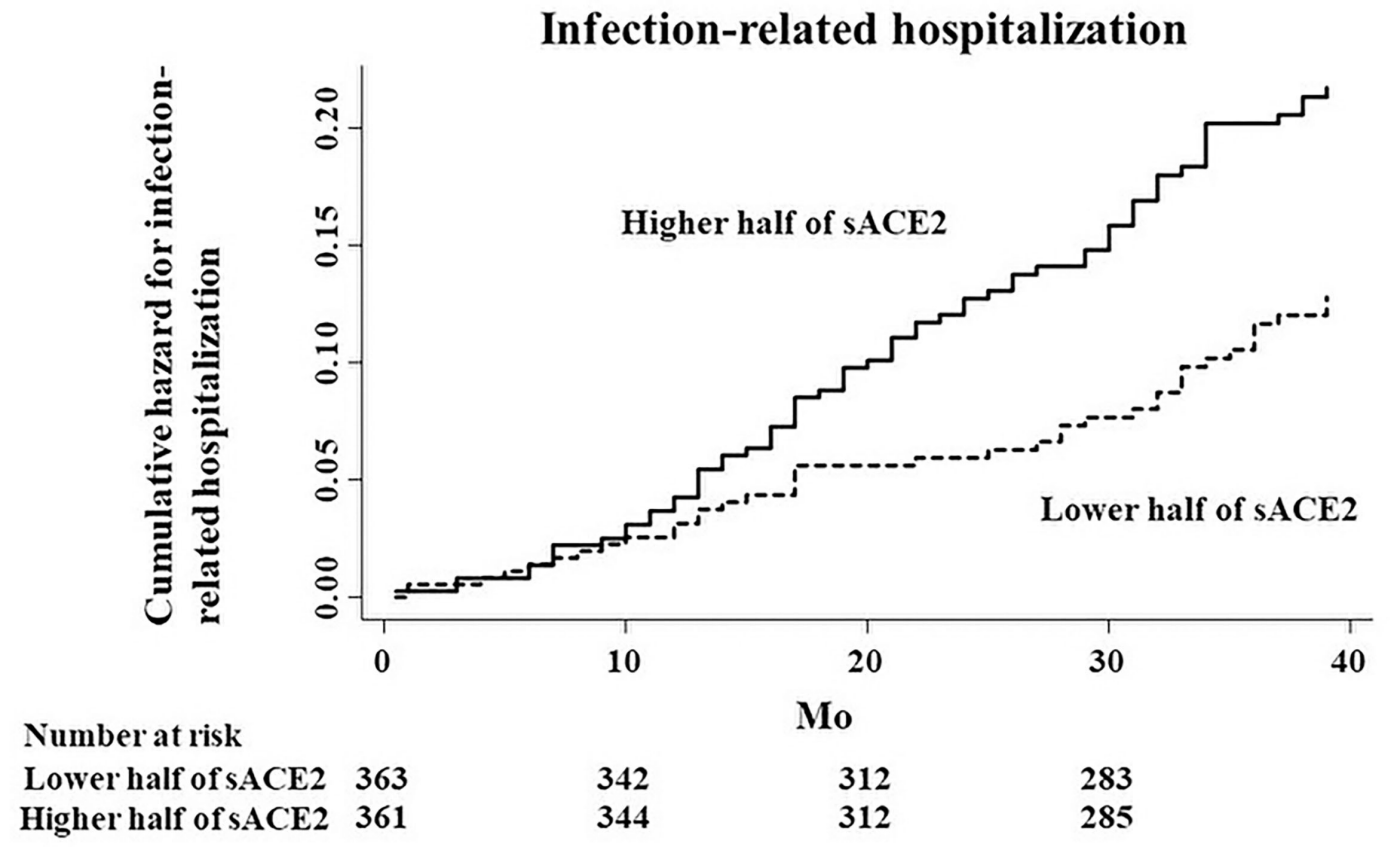
Cumulative hazard curves for infection-related hospitalization by half of the study population according to serum ACE2 (sACE2) levels.

During a median follow-up of 39 months, 106 patients (14.6%) were hospitalized due to infectious diseases. The major type of infection was respiratory (*n* = 36; 34.0%), and the other types of infections were as follows: cellulitis (*n* = 18), gastrointestinal (*n* = 17), liver (*n* = 8), vascular access (*n* = 7), renal cyst infection (*n* = 5), genitourinary (*n* = 4), and others (*n* = 11). Compared with the subgroup of patients with the lower half of sACE2 levels, the subgroup of patients with the higher half was associated with an increased risk of infection-related hospitalization in the unadjusted Cox proportional hazards model (HR, 1.72; 95% CI, 1.16–2.55; *p* = 0.007; Figure 2). Of the other covariates, older age, low levels of hemoglobin, albumin, creatinine, the higher half of IL-6, and a past history of CVD were also associated with the risk of infection-related hospitalization in the univariate Cox proportional hazards model (Table 2). After adjusting by age, sex, dialysis vintage, BMI, and diabetes mellitus as model 1, the higher half of sACE2 remained the significant risk factor for infection-related hospitalization. After adjusting for variables from age to sIL-6R as model 2 and for all variables in model 3, the higher half of sACE2 was the robust risk factor for infection-related hospitalization.

**Table 2:** Univariate and multivariate Cox proportional hazard models of infection-related hospitalization.

**Variable**	**Univariate HR (95% CI)**	***P*-value**	**Multivariate (model 1)** **HR (95% CI)**	***P*-value**	**Multivariate (model 2)** **HR (95% CI)**	***P*-value**	**Multivariate (model 3)** **HR (95% CI)**	***P*-value**
Lower half of sACE2	1.00 (reference)	0.007	1.00 (reference)	0.002	1.00 (reference)	0.03	1.00 (reference)	0.04
Higher half of sACE2	1.72 (1.16–2.55)		1.88 (1.26–2.80)		1.60 (1.05–2.46)		1.57 (1.02–2.41)	
Age	1.03 (1.01–1.05)	0.001	1.03 (1.01–1.05)	0.001	1.02 (1.00–1.04)	0.09	1.02 (0.99–1.04)	0.14
Sex (male)	1.01 (0.67–1.53)	0.95	1.02 (0.67–1.56)	0.91	1.15 (0.69–1.91)	0.59	1.13 (0.67–1.91)	0.65
Dialysis vintage	0.99 (0.97–1.02)	0.69	1.00 (0.97–1.03)	0.93	1.00 (0.97–1.03)	0.97	0.99 (0.96–1.03)	0.74
Body mass index	0.97 (0.92–1.01)	0.17	0.98 (0.92–1.03)	0.36	0.99 (0.93–1.05)	0.65	0.99 (0.93–1.05)	0.75
Diabetes mellitus	1.34 (0.92–1.97)	0.13	1.31 (0.85–2.00)	0.22	1.19 (0.74–1.93)	0.48	1.02 (0.62–1.69)	0.93
Hemoglobin	0.75 (0.63–0.91)	0.003			0.86 (0.69–1.08)	0.19	0.87 (0.70–1.10)	0.24
Albumin	0.44 (0.25–0.76)	0.003			1.03 (0.51–2.07)	0.94	0.97 (0.47–1.98)	0.93
Creatinine	0.91 (0.85–0.97)	0.003			0.95 (0.86–1.04)	0.29	0.96 (0.87–1.06)	0.37
Kt/V	1.20 (0.59–2.44)	0.61			1.44 (0.61–3.43)	0.41	1.52 (0.63–3.64)	0.35
Lower half of IL-6	1.00 (reference)	0.02			1.00 (reference)	0.08	1.00 (reference)	0.21
Higher half of IL-6	1.66 (1.10–2.50)				1.49 (0.95–2.33)		1.34 (0.84–2.12)	
Lower half of sIL-6R	1.00 (reference)	0.38			1.00 (reference)	0.43	1.00 (reference)	0.50
Higher half of sIL-6R	0.84 (0.56–1.24)				0.84 (0.55–1.29)		0.86 (0.56–1.33)	
Past history of CVD	1.82 (1.19–2.79)	0.006					1.54 (0.93–2.56)	0.09
Past history of malignancy	1.18 (0.71–1.96)	0.52					1.05 (0.59–1.88)	0.86
ACE-I or ARB	1.00 (0.68–1.47)	0.99					1.09 (0.70–1.68)	0.71
β blocker	1.48 (0.98–2.23)	0.06					1.37 (0.86–2.20)	0.18

*HR, hazard ratio; CI, confidence interval; sACE2, soluble angiotensin-converting enzyme 2; IL-6, Interleukin-6; sIL-6R, soluble interleukin-6 receptor; CVD, cardiovascular disease; ACE-I, angiotensin-converting enzyme inhibitor; ARB, angiotensin II receptor blocker*.

**Figure 3:**
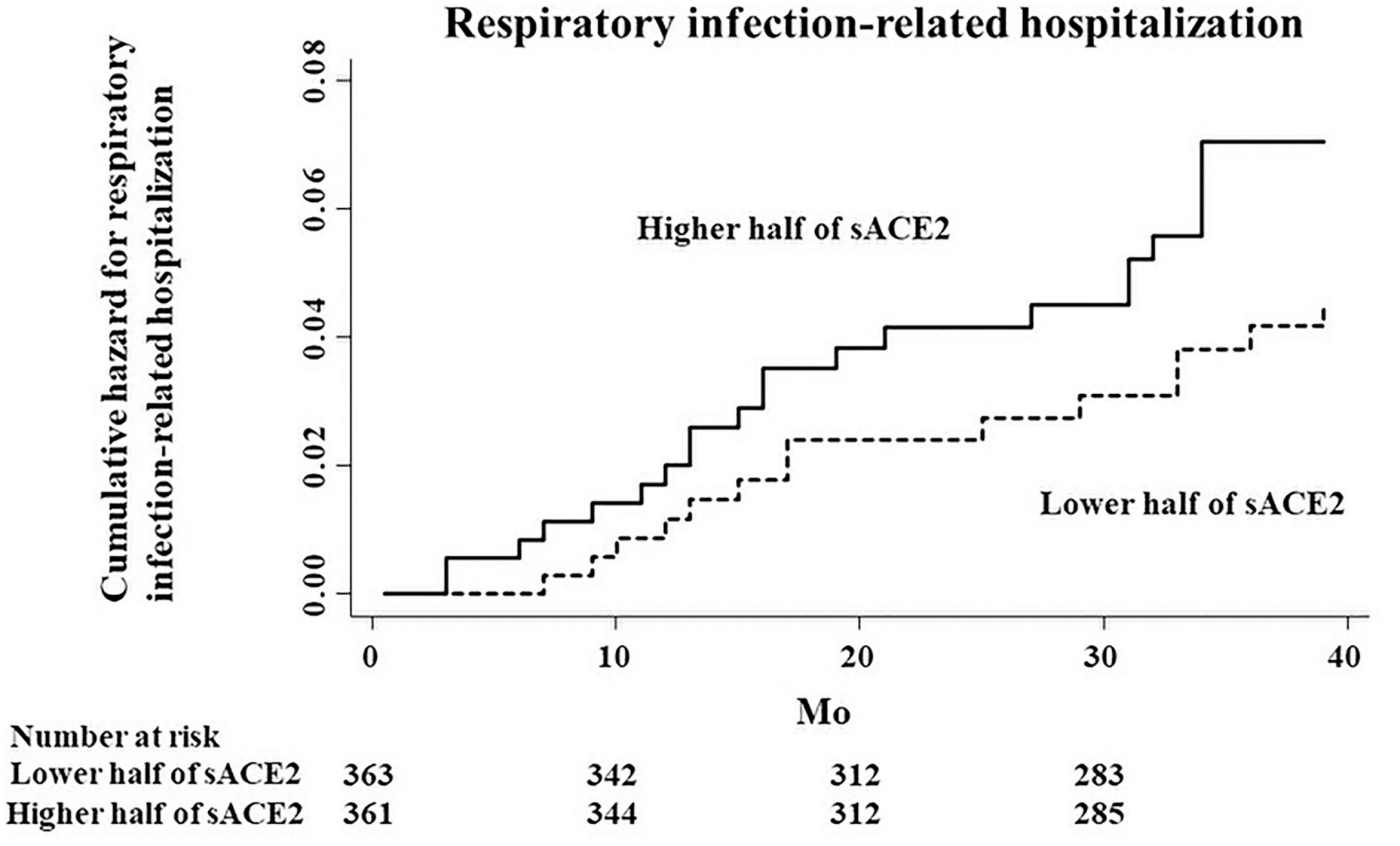
Cumulative hazard curves for respiratory infection-related hospitalization by half of the study population according to serum ACE2 (sACE2) levels.

Restricting to hospitalization due to respiratory infection, the higher half of sACE2 was also a risk factor for infection-related hospitalization, although there was no significant association between sACE2 and hospitalization due to respiratory infection (HR, 1.57; 95% CI, 0.80–3.07; *p* = 0.19; Figure 3).

### Relationship Between sACE2 Levels and All-Cause Death or CVD

A total of 138 patients (19.1%) died during the study period. The distribution of causes was as follows: CVD (*n* = 71), infections (*n* = 29), malignancy (*n* = 24), and others (*n* = 14). The risk of all-cause death was significantly associated with older age, low BMI, diabetes mellitus, low levels of hemoglobin, albumin, and creatinine, the higher half of IL-6, past history of CVD, and malignancy in the unadjusted Cox proportional hazards model (Table 3). There were no significant associations between sACE2 and all-cause death in the unadjusted model (HR, 0.79; 95% CI, 0.57–1.11; *p* = 0.17) and each adjusted model (e.g., model 3; HR, 0.71; 95% CI, 0.48 −1.04; *p* = 0.08).

**Table 3:** Univariate and multivariate Cox proportional hazard models of all-cause death.

**Variable**	**Univariate HR (95% CI)**	***P*-value**	**Multivariate (model 1)** **HR (95% CI)**	***P*-value**	**Multivariate (model 2)** **HR (95% CI)**	***P*-value**	**Multivariate (model 3)** ** HR (95% CI)**	***P*-value**
Lower half of sACE2	1.00 (reference)	0.17	1.00 (reference)	0.49	1.00 (reference)	0.09	1.00 (reference)	0.08
Higher half of sACE2	0.79 (0.57–1.11)		0.89 (0.63–1.25)		0.72 (0.49–1.05)		0.71 (0.48–1.04)	
Age	1.06 (1.04–1.08)	<0.001	1.06 (1.04–1.08)	<0.001	1.05 (1.03–1.07)	<0.001	1.05 (1.02–1.07)	<0.001
Sex (male)	1.38 (0.94–2.03)	0.10	1.47 (0.99–2.19)	0.06	1.86 (1.15–2.99)	0.01	1.76 (1.08–2.86)	0.02
Dialysis vintage	1.00 (0.98–1.02)	0.95	1.01 (0.99–1.04)	0.31	1.01 (0.98–1.04)	0.40	1.00 (0.98–1.03)	0.80
Body mass index	0.94 (0.90–0.98)	0.008	0.95 (0.90–1.01)	0.08	0.98 (0.93–1.04)	0.59	0.99 (0.93–1.05)	0.71
Diabetes mellitus	1.75 (1.25–2.45)	0.001	1.96 (1.35–2.85)	<0.001	1.87 (1.22–2.85)	0.004	1.77 (1.14–2.74)	0.01
Hemoglobin	0.71 (0.60–0.83)	<0.001			0.80 (0.65–0.98)	0.04	0.83 (0.67–1.02)	0.08
Albumin	0.35 (0.21–0.56)	<0.001			1.02 (0.54–1.93)	0.95	0.99 (0.51–1.90)	0.97
Creatinine	0.83 (0.78–0.88)	<0.001			0.88 (0.80–0.96)	0.003	0.88 (0.80–0.96)	0.005
Kt/V	0.78 (0.42–1.45)	0.43			0.79 (0.37–1.68)	0.54	0.86 (0.41–1.80)	0.68
Lower half of IL-6	1.00 (reference)	0.003			1.00 (reference)	0.07	1.00 (reference)	0.15
Higher half of IL-6	1.74 (1.21–2.49)				1.45 (0.97–2.17)		1.36 (0.90–2.08)	
Lower half of sIL-6R	1.00 (reference)	1.00			1.00 (reference)	0.58	1.00 (reference)	0.86
Higher half of sIL-6R	1.00 (0.71–1.40)				1.12 (0.76–1.63)		1.03 (0.70–1.53)	
Past history of CVD	1.98 (1.37–2.85)	<0.001					1.57 (1.03–2.38)	0.04
Past history of malignancy	2.39 (1.65–3.45)	<0.001					2.03 (1.32–3.11)	0.001
ACE-I or ARB	1.01 (0.72–1.41)	0.98					0.92 (0.62–1.37)	0.69
β blocker	1.33 (0.93–1.91)	0.12					1.13 (0.74–1.73)	0.57

*HR, hazard ratio; CI, confidence interval; sACE2, soluble angiotensin-converting enzyme 2; IL-6, Interleukin-6; sIL-6R, soluble Interleukin-6 receptor; CVD, cardiovascular disease; ACE-I, angiotensin-converting enzyme inhibitor; ARB, angiotensin II receptor blocker*.

CVD events occurred in 236 patients (32.6%) during the study period. CVD was significantly associated with older age, diabetes mellitus, low levels of hemoglobin, creatinine, the higher half of IL-6, past history of CVD, use of ACE-Is or ARBs, and use of β blockers in the unadjusted Cox proportional hazards model (Table 4). After adjustment for covariates in model 3, the factors significantly associated with an increased risk of CVD were older age, diabetes mellitus, low levels of creatinine, and past history of CVD. No significant association was observed between the sACE2 level and CVD (HR, 0.83; 95% CI, 0.63–1.11; *p* = 0.20).

**Table 4:** Univariate and multivariate Cox proportional hazard models of cardiovascular disease.

**Variable**	**Univariate HR (95% CI)**	***P*-value**	**Multivariate (model 1)** **HR (95% CI)**	***P*-value**	**Multivariate (model 2)** **HR (95% CI)**	***P*-value**	**Multivariate (model 3)** ** HR (95% CI)**	***P*-value**
Lower half of sACE2	1.00 (reference)	0.21	1.00 (reference)	0.42	1.00 (reference)	0.25	1.00 (reference)	0.20
Higher half of sACE2	0.85 (0.66–1.10)		0.90 (0.69–1.16)		0.85 (0.64–1.12)		0.83 (0.63–1.11)	
Age	1.03 (1.01–1.04)	<0.001	1.03 (1.01–1.04)	<0.001	1.02 (1.01–1.04)	0.003	1.02 (1.01–1.04)	0.005
Sex (male)	1.20 (0.90–1.59)	0.22	1.14 (0.86–1.53)	0.36	1.33 (0.94–1.89)	0.11	1.28 (0.89–1.84)	0.18
Dialysis vintage	0.99 (0.98–1.01)	0.48	1.02 (1.00–1.04)	0.08	1.02 (1.00–1.04)	0.08	1.01 (0.99–1.03)	0.30
Body mass index	1.01 (0.98–1.04)	0.37	1.01 (0.98–1.05)	0.44	1.01 (0.97–1.05)	0.76	1.01 (0.97–1.05)	0.75
Diabetes mellitus	2.10 (1.62–2.71)	<0.001	2.23 (1.67–2.99)	<0.001	2.28 (1.64–3.15)	<0.001	1.96 (1.39–2.75)	<0.001
Hemoglobin	0.86 (0.76–0.98)	0.02			0.92 (0.79–1.07)	0.26	0.92 (0.79–1.07)	0.28
Albumin	0.75 (0.52–1.10)	0.14			1.18 (0.73–1.90)	0.50	1.21 (0.74–1.98)	0.44
Creatinine	0.94 (0.90–0.98)	0.004			0.93 (0.87–0.99)	0.02	0.93 (0.87–0.99)	0.03
Kt/V	0.78 (0.49–1.25)	0.30			1.01 (0.57–1.81)	0.96	1.08 (0.60–1.94)	0.81
Lower half of IL-6	1.00 (reference)	0.04			1.00 (reference)	0.19	1.00 (reference)	0.46
Higher half of IL-6	1.34 (1.02–1.75)				1.21 (0.91–1.63)		1.12 (0.83–1.52)	
Lower half of sIL-6R	1.00 (reference)	0.27			1.00 (reference)	0.08	1.00 (reference)	0.09
Higher half of sIL-6R	1.16 (0.89–1.50)				1.29 (0.97–1.73)		1.29 (0.96–1.74)	
Past history of CVD	2.32 (1.75–3.07)	<0.001					1.68 (1.22–2.32)	0.002
Past history of malignancy	1.03 (0.72–1.47)	0.86					1.01 (0.68–1.50)	0.95
ACE-I or ARB	1.31 (1.01–1.70)	0.04					1.01 (0.75–1.35)	0.96
β blocker	1.50 (1.14–1.97)	0.004					1.34 (0.98–1.83)	0.07

*HR, hazard ratio; CI, confidence interval; sACE2, soluble angiotensin-converting enzyme 2; IL-6, Interleukin-6; sIL-6R, soluble Interleukin-6 receptor; CVD, cardiovascular disease; ACE-I, angiotensin-converting enzyme inhibitor; ARB, angiotensin II receptor blocker*.

## Discussion

In this *post-hoc* analysis of a prospective cohort study, the primary finding was that higher levels of sACE2 were associated with a greater risk of infection-related hospitalization in patients on maintenance HD, which remained significant even after adjustment by multivariate analysis. In CVD, targeted disruption of ACE2 in mice resulted in a severe cardiac contractility defect (19). In contrast, sACE2 activity was shown to be increased in patients with heart failure and correlated with disease severity (20), in which authors hypothesized that cleavage of mACE2 and shedding as sACE2 into the blood circulation through the pathological up-regulation of ADAM17 results in a relative decrease in mACE2 levels and cardiac dysfunction. Patel et al. supported this hypothesis with the data from experimental mice that showed that continuous infusions of angiotensin II suppressed mACE2 levels in the heart by increasing ADAM17 activity with increased plasma ACE2 activity (21). Similar to the pathophysiology of ACE2 in CVD, reduced mACE2 levels were observed in an impaired kidney animal model (22) and in diabetic patients with CKD (23). In addition, high ADAM17 mRNA expression was significantly associated with interstitial fibrosis and increased serum creatinine levels in patients with glomerulonephritis with or without impaired kidney function (24). The activation of the renin-angiotensin system has been well documented in HD patients (25, 26). Taken together, high serum sACE2 levels were hypothesized to be associated with increased ADAM17 activity, reduced mACE2 levels, a reduced anti-inflammatory response, and an increased risk of infection-related hospitalization in patients on maintenance HD.

Restricted to hospitalization due to respiratory infection, there was no significant association, probably because the number of the events was too small to detect a significant association. In experimental models, loss of mACE2 worsened acid and sepsis-induced acute lung failure with a significant increase of angiotensin II, whereas recombinant human ACE2 (rhACE2) could decrease the degree of lung damage (27). In another bacterial infection model, a pre-existing and persistent deficiency of mACE2 led to excessive neutrophil accumulation that was reduced by rhACE2 (28). In a clinical phase II trial, rhACE2 has already been shown to have an acceptable safety profile, and angiotensin II levels decreased rapidly with increasing angiotensin 1–7 levels (29). Furthermore, a recent case report of a patient with severe COVID-19 showed successful treatment with rhACE2 (30). From these laboratory and clinical studies, rhACE2 might work well for infectious diseases including pneumonia. In contrast, the present findings regarding sACE2 levels were the opposite results, suggesting that the majority of sACE2 protein measured by ELISA in the present study may not have enzymatic activity, and that higher levels of sACE2 in serum may be just a mirror image of increased ADAM17 activity and decreased mACE2 levels. On the other hand, it is well-known that ACE2 has renin-angiotensin system-independent functions, such as regulation of intestinal amino acid homeostasis and the gut microbiome (31). Thus, further research is needed to understand the pathophysiology of higher levels of sACE2 in the serum of patients on HD and the association with immune systems.

In contrast, of the secondary outcomes, high sACE2 levels were not significantly associated with all-cause death and CVD in HD patients. A recent large observational study reported that increased plasma ACE2 levels were associated with an increased risk of all-cause death, as well as cardiovascular and non-cardiovascular deaths, in general populations (7). However, in two previous studies of CKD including dialysis, baseline circulating ACE2 was not associated with all-cause mortality (32, 33). The present results were consistent with these results. In CKD, Anguiano et al. reported that higher baseline circulating ACE2 levels were associated with an increased number of territories with plaques and the ankle-brachial index, but not with all-cause mortality (33). This is likely because most all-cause deaths in dialysis patients are CVD due to left ventricular hypertrophy, as well as non-traditional risk factors, such as chronic volume overload, anemia, inflammation, oxidative stress, CKD-mineral bone disorder, and uremic factors (34–36). Many previous studies, including a recent large observational study, have suggested that plasma IL-6 was an independent predictor of mortality, not only in all CKD strata, but also in dialysis patients, reflecting chronic inflammation (37–40). Similarly, in the present study, not sACE2 levels, but higher IL-6 levels were significantly associated with an increased risk of all-cause death and CVD.

In the present study, logarithm-transformed sACE2 levels showed a weak negative association with age, but not with sex, BMI, diabetes mellitus, and use of anti-hypertensive medicines such as ACE-Is or ARBs. Previous CKD studies showed that male sex and advanced age were independent predictors of circulating ACE2 activity (32, 41). In contrast to these studies, logarithm-transformed sACE2 levels were not found to be higher in male or older patients in the present study. Given that the gene encoding ACE2 is located on the X chromosome, X chromosome inactivation escape might also play a part in observed differences in ACE2 between men and women, whereas a subgroup analysis of a large observational study suggested that there was little evidence for a heterogeneous effect of ACE2 between the sexes (7). Furthermore, because the present study involved a single ethnic group, ancestral groups or genetic variants might affect the results.

This analysis had several limitations. First, because this was an observational study, causal relationships between sACE2 levels and the primary and secondary outcomes could not be inferred. Second, only serum sACE2 levels were measured; mACE2 protein expression was not investigated. Third, sACE2 requires special consideration with regard to biological interpretation. Although increased mACE2 exerts protective effects against vasoconstriction, oxidative damage, and inflammation, the mechanism by which serum levels rise remains an area of active research. It is likely that a complex interaction among cellular expression, enzymatic cleavage, and impaired serum clearance affects serum concentrations. Fourth, serum sACE2 levels were measured only at baseline, and thus trends in sACE2 levels over time were not assessed in this study population. Further research is needed to examine whether changes of sACE2 levels may play a role in subsequent outcomes or time to event. Fifth, the serum levels of ACE, angiotensinogen, angiotensin 1–7, chymase, and the other enzymes of the renin-angiotensin system were not measured in the patients of this cohort, and thus their associations with the risk of infection were not evaluated. Sixth, serum sACE2 levels of healthy patients as controls were not measured. Seventh, this study was conducted in Japan. Patients with end-stage kidney disease often undergo HD in Japan, but kidney transplantation tends to be prioritized in Europe and the United States. Thus, the results of the present study are not necessarily generalizable to other populations. Eighth, the standard error of median was not used in this study, because non-normal continuous variables are usually expressed by medians and IQR.

In conclusion, in patients on maintenance HD, high sACE2 levels showed an independent association with an increased risk of infection-related hospitalization, whereas they were not associated with all-cause death and CVD. The relationship between sACE2 levels and mACE2 expression was not investigated, but the results indicated the possibility of sACE2 as a biomarker to predict severe infectious diseases. Remarkably, sACE2 was only associated with age, but not with sex, BMI, diabetes mellitus, and use of ACE-Is or ARBs. Modulation of ACE2 and the counter-balancing arm might have an important role, and further studies are needed to understand the role of mACE2 and sACE2 in patients on maintenance HD.

## Data Availability Statement

The raw data supporting the conclusions of this article will be made available by the authors, without undue reservation.

## Ethics Statement

The studies involving human participants were reviewed and approved by the Ethics Committee of The Jikei University School of Medicine [Ethics Approval Code: 22-182(6359)]. The patients/participants provided their written informed consent to participate in this study.

## Author Contributions

MK and MU were responsible for the study conception, design, and data analysis and drafted and revised the manuscript. AN was responsible for data acquisition. IY, IO, and TY were responsible for supervision. All authors provided input for the final version of the manuscript.

## Funding

This research was supported by the Jikei University Research Fund for Graduate Students and by The Jikei University School of Medicine.

## Conflict of Interest

The authors declare that the research was conducted in the absence of any commercial or financial relationships that could be construed as a potential conflict of interest.

## Publisher's Note

All claims expressed in this article are solely those of the authors and do not necessarily represent those of their affiliated organizations, or those of the publisher, the editors and the reviewers. Any product that may be evaluated in this article, or claim that may be made by its manufacturer, is not guaranteed or endorsed by the publisher.
